# Cardiometabolic multimorbidity in relation to the metabolic score for insulin resistance and creatinine-to-cystatin C ratio in a middle-aged and aged population

**DOI:** 10.3389/fendo.2025.1694959

**Published:** 2025-12-01

**Authors:** Roushan Zhang, Jian Ma, Li Wang

**Affiliations:** 1 Department of Geriatric Medicine, The Second Affiliated Hospital of Chongqing Medical University, Chongqing, China; 2 Department of Cardiology, The Second Affiliated Hospital of Chongqing Medical University, Chongqing, China

**Keywords:** cardiometabolic multimorbidity, METS-IR, CCR, MRII, CHARLS (ChinaHealth and Retirement Longitudinal Study)

## Abstract

**Objective:**

With the aging population, cardiometabolic multimorbidity (CMM) has become a major public health concern, increasing disease burden and impairing quality of life. The metabolic score for insulin resistance (METS-IR) and creatinine-to-cystatin C (CCR) are promising biomarkers linked to metabolic dysfunction and muscle-renal status, respectively. However, their combined effects on cardiometabolic multimorbidity (CMM), especially in both community and hospitalized populations, remain unclear. This study aims to explore the associations of METS-IR, CCR, and 1/CCR×METS-IR (MRII) with CMM using data from the China Health and Retirement Longitudinal Study (CHARLS) and clinical sources.

**Research design and methods:**

This cross-sectional study included 10,811 participants from the 2014–2015 CHARLS follow-up and 437 elderly inpatients from the Second Affiliated Hospital of Chongqing Medical University. CMM was defined as the coexistence of two or more of heart disease, diabetes, and stroke. METS-IR and CCR were calculated using standard formulas. Logistic regression analyses with multi-model adjustment, restricted cubic spline (RCS) curves, receiver operating characteristic (ROC) curves, and subgroup analyses were performed to assess associations, nonlinear relationships, predictive value, and effect modification.

**Results:**

In both datasets, participants with CMM had higher METS-IR, older age, and higher prevalence of metabolic risk factors. METS-IR was independently and dose-dependently associated with increased CMM risk. CCR showed context-dependent associations, with inverse links in partially adjusted CHARLS models but no significance in clinical data. The “Low CCR and High METS-IR” combination and highest quartile of MRII were consistently linked to elevated CMM risk. METS-IR had moderate predictive value (AUC = 0.712 in CHARLS, 0.618 in clinical data), outperforming CCR. RCS curves revealed linear associations for METS-IR and U-shaped patterns for CCR in CHARLS. Subgroup analyses showed heterogeneity by age, comorbidities, and hypertension.

**Conclusion:**

METS-IR is a robust independent predictor of CMM in both community and hospitalized populations, while CCR’s role is context-dependent. The MRII enhances CMM risk stratification, highlighting the value of concurrent assessment of metabolic and muscle-renal status for CMM prevention and personalized risk management.

## 1 Introduction

The aging population has led to a significant increase in chronic diseases, which in turn has elevated the prevalence of multimorbidity (1, 2). Multimorbidity is generally defined as the coexistence of two or more chronic diseases or disease groups (2). Compared with single cardiovascular diseases, multimorbidity exerts more adverse effects on human health (3). Cardiometabolic multimorbidity (CMM), defined as having two or more physician-diagnosed conditions like heart diseases (e.g., myocardial infarction, coronary heart disease, angina pectoris, congestive heart failure, or other cardiac disorders), diabetes mellitus (including impaired glucose tolerance and elevated fasting blood glucose), and stroke, exerts severe adverse impacts on human health (4, 5). Existing research has explored the influence of long-term air pollution exposure on CMM (6), as well as the links between fat-related indices such as the triglyceride-glucose index (TyG) and lipid accumulation product (LAP) and CMM (3, 7–9). CMM escalates the risks of mortality (10), dementia (11, 12), and depressive symptoms (13). It also impairs lifestyle behaviors (14) and cognitive function (15), etc. Thus, preventing CMM effectively is vital for cutting disease burden and enhancing patients’ quality of life.

The metabolic score for insulin resistance (METS-IR), a clinical surrogate marker for obesity-related insulin resistance (IR), was developed by Mexican researchers to assess insulin sensitivity. Recognized as a more precise way to measure insulin sensitivity, higher METS-IR values signal greater insulin resistance and a heightened risk of metabolic disorders (16). Studies have found an M-shaped association between METS-IR and heart failure in American adults (17), suggesting its potential as a marker for predicting heart failure. A retrospective study in Gifu, Japan, showed METS-IR levels were linked to prehypertension or hypertension in normoglycemic individuals (18). An 8-year longitudinal study revealed a linear dose-response between METS-IR and cardiovascular disease risks (e.g., cardiovascular disease, stroke, heart disease) (19).

Serum creatinine-to-cystatin C(CCR) was used to evaluate renal function. The ratio (serum creatinine/cystatin C multiplied by 100) was validated by Kashani et al. in 2016 for correlating with muscle mass, defined as the “sarcopenia index” (20). Regarded as a reliable marker for assessing muscle mass (21), CCR predicts muscle mass loss and sarcopenia in diseases like diabetes, tumors, and chronic obstructive pulmonary disease (22–25). The relationships between METS-IR, CCR, and cardiovascular diseases have been extensively studied in community populations worldwide. These studies have confirmed that both the CCR and METS-IR are individually associated with CMM in community populations. However, the combined effect of these two indices on CMM, as well as their respective correlations with CMM in hospitalized patients, remains unclear. This study innovatively defines the product inverse of CCR and METS-IR (1/CCR×METS-IR) as the “metabolic-kidney interaction index (MRII)”. The aim of this study is to investigate the impacts of the CCR, METS-IR and MRII on CMM in both community and hospitalized populations in China.

## 2 Materials and methods

### 2.1 Study population

**Figure 1:**
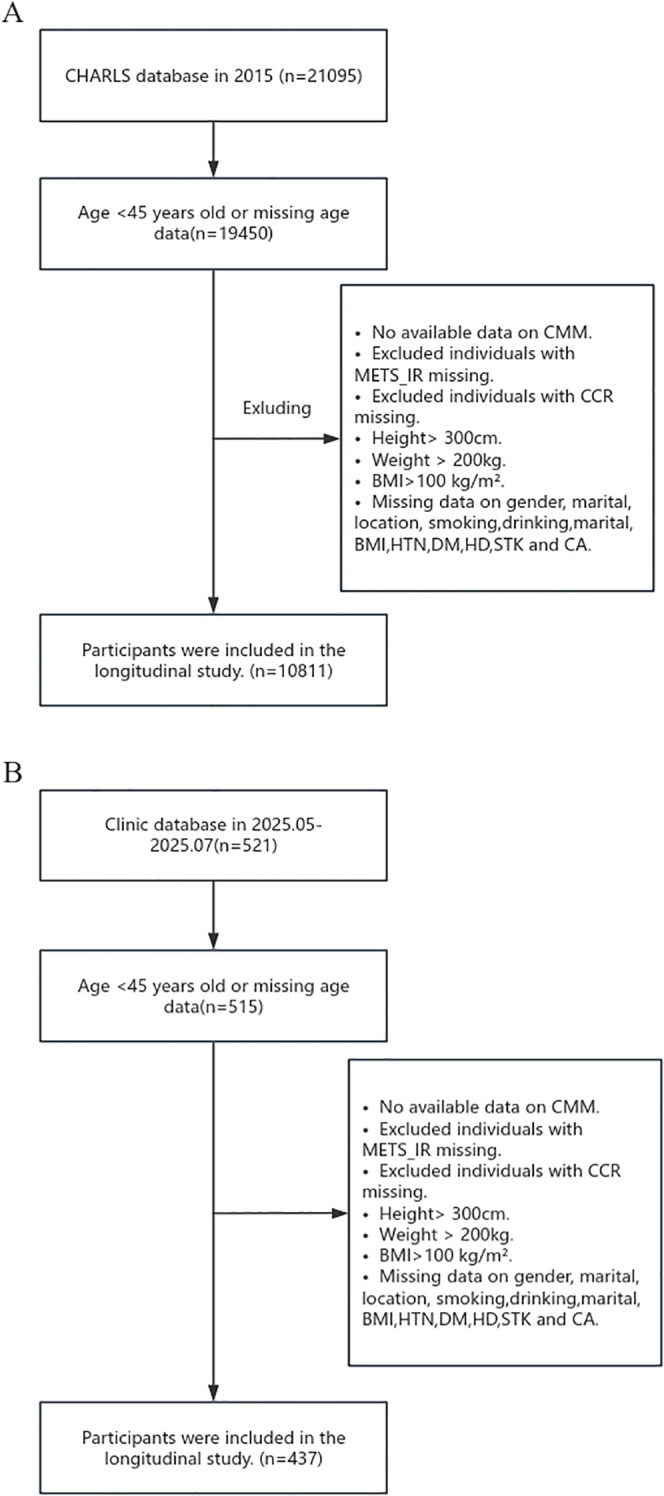
Flow Charts. **(A)** Flow chart of CHARLS participants. **(B)** Flow chart of clinical participants.

This study incorporated data from both the China Health and Retirement Longitudinal Study (CHARLS) database and clinical sources. The CHARLS, spearheaded by the National School of Development at Peking University, is designed to evaluate the health, socioeconomic, and demographic characteristics of Chinese adults aged 45 and over. Spanning 28 provinces, it uses a multi-stage, stratified, and clustered sampling approach. Follow-up surveys were carried out in 2013, 2015, 2018, and 2020. Ethical approval was granted by the Biomedical Ethics Review Committee of Peking University (RBK00001052-11015), and all participants provided informed consent. The data can be accessed online at http://charls.pku.edu.cn. The research methodology and data collection processes for CHARLS have been elaborated in existing literature. For this study, the third-wave follow-up data (2014–2015) were chosen, initially including 20,967 participants. After excluding those with missing data and outliers, 10,811 participants remained in the final analysis. Regarding clinical data, 521 middle-aged and elderly patients admitted to the Department of Geriatrics at the Second Affiliated Hospital of Chongqing Medical University were enrolled between May and July, 2025. After excluding participants with incomplete data, 437 clinical patients were included in the final analysis. The inclusion and exclusion criteria for both samples are presented in **Figures 1A**, **B**, respectively.

### 2.2 Assessment of CMM events

Cardiometabolic multimorbidity (CMM) is defined as the coexistence of two or more cardiovascular-related diseases, including heart disease, diabetes, and stroke.

For the CHARLS database, the diagnosis of heart disease was determined based on questionnaire responses indicating that participants had been diagnosed by a doctor with conditions such as heart failure, coronary heart disease, myocardial infarction, or other heart diseases, or were taking heart disease-related medications. Diabetes was defined as a self-reported physician’s diagnosis or the presence of any one of the following biochemical criteria: fasting blood glucose ≥ 7.8 mmol/L, HbA1c ≥ 6.0%, or random blood glucose ≥ 11.1 mmol/L. Additionally, individuals taking diabetes-related medications or receiving insulin injections were also classified as having diabetes. Stroke was identified based on either a participant’s report of a physician-diagnosed cerebrovascular event (such as cerebral infarction or cerebral hemorrhage) or the use of antithrombotic or other stroke-specific medications. In the clinical data, heart disease was primarily defined by discharge diagnoses including coronary heart disease, heart failure, myocardial infarction, or other heart diseases. Diabetes was determined based on discharge diagnoses of type 1 or type 2 diabetes. Stroke was identified by discharge diagnoses of cerebrovascular accidents such as cerebral infarction or cerebral hemorrhage.

### 2.3 Assessment of METS-IR​

The metabolic score for insulin resistance (METS-IR) has become a promising indirect approach to identify insulin resistance (IR) related to the pathophysiological elements of metabolic syndrome (26). Current research has indicated that METS-IR is associated with conditions like diabetes, hypertension, obstructive sleep apnea, and kidney stones (27–30). Instead of directly measuring insulin, METS-IR evaluates IR using body mass index (BMI), triglycerides (TG), and fasting plasma glucose (FPG), which makes it highly suitable for large-scale screening and clinical application. As an indicator of insulin resistance, a higher METS-IR value means a more severe degree of insulin resistance, implying that the individual faces a greater likelihood of developing metabolic disorders. The formula for calculating METS-IR is: METS-IR = Ln [(2*FPG) + TG] × BMI (kg/m²) ÷ (Ln[HDL-C]). Here, FPG stands for fasting plasma glucose (mg/dL), HDL-C is high-density lipoprotein cholesterol (mg/dL), and TG represents triglycerides (mg/dL). In the above formula, FPG, HDL-C and TG represent fasting plasma glucose (mg/dL), high-density lipoprotein cholesterol (mg/dL), and triglycerides (mg/dL), respectively.

### 2.4 Assessment of CCR​

The creatinine-to-cystatin C ratio (CCR) is an indicator used to evaluate renal function. By comparing the concentrations of serum creatinine and cystatin C, it helps assess glomerular filtration rate (GFR) (31–34). Serum creatinine varies with body composition, while cystatin C is widely present in nucleated cells and is less affected by muscle mass (35). Thus, CCR can provide information on an individual’s muscle mass and renal function and serves as a risk factor for sarcopenia. The calculation formula of CCR is: CCR = creatinine (mg/dL)/cystatin C (mg/L) × 100.

### 2.5 Covariates

Covariates included demographic and health-related variables. Demographic characteristics encompassed age, gender, geographical residence, and marital status. Health-related indicators consisted of anthropometric parameters (height, weight, and BMI), as well as medical history (stroke, heart disease, diabetes, hypertension, chronic lung disease, and malignant tumors). The formula for calculating BMI is: weight (kg) divided by the square of height (m²).

Blood test data from the CHARLS database were centrally analyzed by the Youanmen Clinical Laboratory Center of Capital Medical University using the enzyme colorimetric method. Two staff members from the Chinese Center for Disease Control and Prevention were fully responsible for the storage of blood samples. During the testing process, the laboratory used quality control samples daily, with the intra-batch coefficient of variation not exceeding 1.0% and the inter-batch coefficient of variation not exceeding 1.7%. Lipid profiles including total cholesterol (TC), low-density lipoprotein cholesterol (LDL-C), high-density lipoprotein cholesterol (HDL-C), and triglycerides (TG) were quantified using enzymatic assays, while high-sensitivity C-reactive protein (hsCRP) concentrations were measured by immunoturbidimetry. The definitions of hypertension, diabetes, and stroke were as described above, and the definitions of other chronic diseases were determined by self-report. Laboratory parameters from the clinical cohort were obtained from fasting blood samples sent to the hospital laboratory on the day of admission or the morning of the second day after admission. Chronic disease data in the clinical data were the diseases included in the discharge diagnosis.

## 3 Statistical analysis

The analysis of baseline data consists of two core parts: descriptive statistics and inter-group comparison tests. For descriptive statistics, appropriate statistical measures are selected based on the type and distribution characteristics of variables. Normally distributed continuous variables are described using mean ± standard deviation (x̄ ± s). Skewed continuous variables are presented as median (interquartile range) [M (P25, P75)]. Categorical variables are expressed as frequency (percentage) [n (%)]. For inter-group comparisons, corresponding statistical tests are applied according to variable types and distribution characteristics. The chi-square test is used for comparing categorical variables, and Fisher’s exact test is adopted when the theoretical frequency is less than 5. For continuous variables, the independent samples t-test is used if they are normally distributed with homogeneous variances, otherwise the Mann-Whitney U test is employed.

To ensure model robustness and avoid interference from spurious or collinear variables, we applied two key screening criteria: (1) only variables with a statistical significance level of P ≤ 0.001 were retained; (2) variables that would induce severe multicollinearity were excluded. Subsequently, the screened variables were merged with basic confounding variables to form an initial variable pool for model construction. Finally, bidirectional stepwise regression was used to iteratively optimize and determine the final variables included in Models 1–4 for each dataset separately. To clarify the association between CCR, METS-IR and CMM, this study uses multi-model logistic regression analysis. First, CCR and METS-IR are divided into 3 levels (Q1, Q2, Q3) by the tertile method, with the lowest tertile group (Q1) as the reference group, to evaluate the strength of association between different groups and the risk of CMM. Based on differences in data sources, the following regression models are constructed respectively: For the CHARLS database: Model 1 is unadjusted for any covariates; Model 2 is adjusted for age and gender; Model 3 is further adjusted for location, marital status, smoking history, drinking history, hypertension (HTN), chronic lung disease (CLD), and cancer (CA) on the basis of Model 2; Model 4 is additionally adjusted for white blood cell count (WBC), glycosylated hemoglobin (HbA1c), and total cholesterol (TC) on the basis of Model 3. For the clinical database (Clinic data): Model 1 is unadjusted for any covariates; Model 2 is adjusted for age and gender; Model 3 is further adjusted for location, marital status, and hypertension (HTN) on the basis of Model 2; Model 4 is additionally adjusted for white blood cell count (WBC) and low-density lipoprotein cholesterol (LDL-C) on the basis of Model 3.

This study employs the MRII metric to examine its correlation with CMM, rather than using the standard interaction term (CCR×METS-IR) for analysis. Biologically, 1/CCR aligns CCR’s negative association with CMM with METS-IR’s positive association (36–38), enabling intuitive interpretation. Meanwhile, the Vuong test revealed that the MRII model outperformed the CCR×METS-IR model significantly in the CHARLS dataset, with statistically significant differences (**Supplementary Tables S1**, **S2**). The MRII is calculated and divided into 4 levels (Q1, Q2, Q3, Q4) by the quartile method. With the lowest quartile group (Q1) as the reference, the same multi-model logistic regression as mentioned above is used to analyze its association with CMM. Meanwhile, restricted cubic spline (RCS) curves are used to analyze the dose-response relationship between CCR, METS-IR and CMM, so as to clarify whether there is a nonlinear association between them; receiver operating characteristic (ROC) curves are plotted and the area under the curve (AUC) is calculated to evaluate the predictive efficacy of CCR, METS-IR and MRII for CMM, and to compare the predictive value of different indicators.

To investigate the impact of different population characteristics on the association between CCR, METS-IR, MRII and CMM, further subgroup analysis is conducted. Stratification is performed according to key variables such as age, gender, and hypertension status.

The analyses employed Empower^®^ version 4.4.3.

## 4 Results

### 4.1 Based on baseline data tables for the occurrence of CMM

This study investigated the associations of the metabolic score for insulin resistance (METS-IR) and the creatinine-to-cystatin C ratio (CCR) with cardiometabolic multimorbidity (CMM) using data from the China Health and Retirement Longitudinal Study (CHARLS) and clinical sources, and baseline data analysis was conducted firstly (**Table 1**). In the CHARLS cohort (n = 10,811), participants with CMM (n = 463) exhibited a higher age (median 66.0 vs. 61.5 years, p < 0.001) and BMI (median 25.8 vs. 23.5 kg/m², p < 0.001) than those without CMM (n = 10,348). The CMM group also had a higher prevalence of hypertension (66.5% vs. 23.5%, p < 0.001), diabetes (92.2% vs. 15.6%, p < 0.001), and a higher METS-IR (41.3 vs. 34.8, p < 0.001). In the clinical data cohort (n = 437), the CMM subgroup (n = 154) had an older median age (75.0 vs. 69.0 years, p < 0.001), higher diabetes prevalence (76.0% vs. 16.3%, p < 0.001), and elevated METS-IR (29.8 vs. 27.9, p < 0.001) relative to the non-CMM group (n = 283).

**Table 1:** Baseline table.

	Level	Overall	Non-CMM	CMM	P	Overall	Non-CMM	CMM	P
N		10811	10348	463		437	283	154	
Age (median [IQR])		61.0 [54.0, 68.0]	61.0 [54.0, 68.0]	66.0 [60.0, 72.0]	<0.001	71.0 [63.0, 79.0]	69.0 [60.5, 77.0]	75.0 [68.0, 82.0]	<0.001
Age_group (%)	<70	8628 (79.8)	8313 (80.3)	315 (68.0)	<0.001	194 (44.4)	143 (50.5)	51 (33.1)	0.001
	≥70	2183 (20.2)	2035 (19.7)	148 (32.0)		243 (55.6)	140 (49.5)	103 (66.9)	
Gender (%)	female	5757 (53.3)	5492 (53.1)	265 (57.2)	0.088	250 (57.2)	168 (59.4)	82 (53.2)	0.257
	male	5054 (46.7)	4856 (46.9)	198 (42.8)		187 (42.8)	115 (40.6)	72 (46.8)	
Marital (%)	married	8891 (82.2)	8513 (82.3)	378 (81.6)	0.778	372 (85.1)	249 (88.0)	123 (79.9)	0.033
	unmarried	1920 (17.8)	1835 (17.7)	85 (18.4)		65 (14.9)	34 (12.0)	31 (20.1)	
Location (%)	city	9677 (89.5)	9313 (90.0)	364 (78.6)	<0.001	363 (83.1)	228 (80.6)	135 (87.7)	0.079
	village	1134 (10.5)	1035 (10.0)	99 (21.4)		74 (16.9)	55 (19.4)	19 (12.3)	
Smoking (%)	no smoking	6299 (58.3)	6019 (58.2)	280 (60.5)	0.348	342 (78.3)	222 (78.4)	120 (77.9)	0.996
	smoking	4512 (41.7)	4329 (41.8)	183 (39.5)		95 (21.7)	61 (21.6)	34 (22.1)	
Drinking (%)	drinking	3735 (34.5)	3626 (35.0)	109 (23.5)	<0.001	73 (16.7)	48 (17.0)	25 (16.2)	0.952
	no drinking	7076 (65.5)	6722 (65.0)	354 (76.5)		364 (83.3)	235 (83.0)	129 (83.8)	
HTN (%)	N	8076 (74.7)	7921 (76.5)	155 (33.5)	<0.001	191 (43.7)	146 (51.6)	45 (29.2)	<0.001
	Y	2735 (25.3)	2427 (23.5)	308 (66.5)		246 (56.3)	137 (48.4)	109 (70.8)	
DM (%)	N	8774 (81.2)	8738 (84.4)	36 (7.8)	<0.001	274 (62.7)	237 (83.7)	37 (24.0)	<0.001
	Y	2037 (18.8)	1610 (15.6)	427 (92.2)		163 (37.3)	46 (16.3)	117 (76.0)	
CLD (%)	N	9561 (88.4)	9191 (88.8)	370 (79.9)	<0.001	381 (87.2)	246 (86.9)	135 (87.7)	0.944
	Y	1250 (11.6)	1157 (11.2)	93 (20.1)		56 (12.8)	37 (13.1)	19 (12.3)	
HD (%)	N	9390 (86.9)	9330 (90.2)	60 (13.0)	<0.001	219 (50.1)	203 (71.7)	16 (10.4)	<0.001
	Y	1421 (13.1)	1018 (9.8)	403 (87.0)		218 (49.9)	80 (28.3)	138 (89.6)	
STK (%)	N	10561 (97.7)	10224 (98.8)	337 (72.8)	<0.001	320 (73.2)	253 (89.4)	67 (43.5)	<0.001
	Y	250 (2.3)	124 (1.2)	126 (27.2)		117 (26.8)	30 (10.6)	87 (56.5)	
CA (%)	N	10685 (98.8)	10237 (98.9)	448 (96.8)	<0.001	423 (96.8)	272 (96.1)	151 (98.1)	0.415
	Y	126 (1.2)	111 (1.1)	15 (3.2)		14 (3.2)	11 (3.9)	3 (1.9)	
BMI (median [IQR])		23.6 [21.2, 26.2]	23.5 [21.2, 26.1]	25.8 [23.2, 28.6]	<0.001	23.6 [21.1, 25.8]	23.4 [20.9, 26.0]	24.1 [21.6, 25.8]	0.134
BMI_group (%)	<24	5827 (53.9)	5687 (55.0)	140 (30.2)	<0.001	241 (55.1)	167 (59.0)	74 (48.1)	0.036
	≥24	4984 (46.1)	4661 (45.0)	323 (69.8)		196 (44.9)	116 (41.0)	80 (51.9)	
HB (median [IQR])		13.6 [12.5, 14.8]	13.6 [12.5, 14.8]	13.6 [12.8, 14.8]	0.056	13.0 [11.9, 14.1]	13.0 [12.0, 14.1]	12.8 [11.7, 13.9]	0.198
WBC (median [IQR])		5.7 [4.8, 6.9]	5.7 [4.7, 6.8]	6.1 [5.3, 7.5]	<0.001	5.6 [4.8, 6.9]	5.4 [4.7, 6.6]	5.8 [5.2, 7.3]	0.002
PLT (median [IQR])		200.0 [159.0, 242.0]	200.0 [159.0, 242.0]	200.0 [158.0, 246.0]	0.566	185.0 [152.0, 226.0]	190.0 [156.5, 229.5]	176.5 [144.2, 207.8]	0.01
FPG (median [IQR])		5.3 [4.9, 5.9]	5.3 [4.9, 5.8]	6.7 [5.5, 8.5]	<0.001	5.4 [4.9, 6.3]	5.2 [4.8, 5.8]	6.0 [5.1, 7.6]	<0.001
TG (median [IQR])		1.3 [0.9, 1.9]	1.3 [0.9, 1.8]	1.7 [1.2, 2.5]	<0.001	1.2 [0.9, 1.7]	1.1 [0.8, 1.6]	1.3 [1.0, 1.8]	0.01
HDL-C (median [IQR])		1.3 [1.1, 1.5]	1.3 [1.1, 1.5]	1.2 [1.0, 1.4]	<0.001	1.3 [1.1, 1.5]	1.4 [1.1, 1.6]	1.2 [1.0, 1.4]	<0.001
TC (median [IQR])		181.5 [159.8, 206.2]	181.5 [159.5, 205.8]	189.6 [164.3, 213.9]	<0.001	172.8 (42.7)	178.2 (46.7)	163.0 (45.2)	0.001
LDL (median [IQR])		100.8 [82.6, 119.7]	100.8 [82.6, 119.6]	103.5 [82.0, 123.4]	0.112	86.2 [66.5, 106.3]	88.9 [71.2, 108.5]	77.0 [61.2, 100.7]	<0.001
UA (median [IQR])		4.8 [3.9, 5.7]	4.8 [3.9, 5.7]	5.1 [4.4, 6.2]	<0.001	5.1 [4.0, 6.2]	5.0 [4.0, 6.0]	5.2 [4.2, 6.6]	0.033
Creatinine (median [IQR])		0.8 [0.7, 0.9]	0.8 [0.7, 0.9]	0.8 [0.6, 0.9]	0.63	0.8 [0.7, 1.0]	0.8 [0.7, 0.9]	0.9 [0.7, 1.1]	<0.001
Cystatin_C (median [IQR])		0.8 [0.7, 1.0]	0.8 [0.7, 0.9]	0.9 [0.8, 1.0]	<0.001	1.0 [0.8, 1.2]	0.9 [0.8, 1.1]	1.1 [0.9, 1.4]	<0.001
HbA1c (median [IQR])		5.8 [5.5, 6.1]	5.8 [5.5, 6.1]	6.7 [6.0, 7.6]	<0.001	6.1 [5.7, 6.5]	5.9 [5.6, 6.2]	6.6 [6.0, 7.5]	<0.001
TyG (median [IQR])		1.2 [0.9, 1.7]	1.2 [0.8, 1.7]	1.8 [1.3, 2.3]	<0.001	1.2 [0.8, 1.6]	1.1 [0.7, 1.5]	1.4 [1.0, 1.7]	<0.001
CCR(median [IQR])		92.0 [80.2, 105.8]	92.3 [80.6, 106.1]	85.8 [73.8, 98.6]	<0.001	82.0 [72.6, 91.0]	82.1 [73.7, 91.0]	81.7 [70.9, 90.7]	0.474
METS-IR (median [IQR])		35.0 [30.3, 40.3]	34.8 [30.2, 40.0]	41.3 [36.1, 47.0]	<0.001	28.3 [25.2, 31.9]	27.7 [24.6, 31.2]	29.6 [26.6, 33.6]	<0.001
eGFR (median [IQR])		92.6 [81.5, 100.0]	92.7 [81.7, 100.0]	89.3 [76.8, 96.5]	<0.001	82.7 [66.3, 92.8]	86.4 [72.2, 95.4]	75.1 [51.3, 86.3]	<0.001

CHARLS, Longitudinal Study on Health and Retirement in China; CMM, cardiometabolic multimorbidity; Non-CMM, participants without cardiometabolic multimorbidity; METS-IR, Metabolic score for insulin resistance; CCR, creatinine-to-cystatin C ratio; HTN, Hypertension; DM, Diabetes; CA, Cancer; CLD, Chronic lung disease; HD, heart disease; STK, Stroke; HB, Hemoglobin (g/dL); WBC, White Blood Cell (in thousands); PLT, Platelets (10^9/L); FPG, Glucose (mmol/L); TG, Triglyceride (mmol/L); HDL-C, High-density lipoprotein cholesterol (mmol/L); TC, total cholesterol (mg/dL); LDL, Low density lipoprotein (mg/dL); UA, Uric acid (mg/dL); HbA1c, Hemoglobin A1c (%); eGFR, Estimated glomerular filtration rate (mL/min/1.73 m^2).

### 4.2 Impacts of CCR, METS-IR, MRII on CMM occurrence

Logistic regression analysis was conducted on the relationship between CCR and CMM based on the CHARLS database (**Table 2.1**). In the analysis of the CHARLS database, the association between CCR_per_TIR and the outcome across sequential models was as follows. Model 1 (unadjusted) yielded an OR of 0.99 (95% CI: 0.99–1.00, P < 0.001). Model 2 yielded an OR of 1.00 (95% CI: 0.99–1.00, P = 0.001) after adjustment for age and gender. Model 3 (further adjusted for location, marital status, smoking, drinking, HTN, CLD, CA) presented an OR of 1.00 (95% CI: 0.99–1.00, P = 0.01). Model 4 (adjusted for age, gender, location, marital, HTN, WBC and LDL) yielded an OR of 1.00 (95% CI:1.00–1.00, P = 0.353). For CCR layering (Q1 as reference), Q2 in Model 1 had an OR of 0.98 (95% CI: 0.97–0.99, P < 0.001), Q3 in Model 1 showed an OR of 0.97 (95% CI: 0.96–0.98, P < 0.001), with varying significances across models. **Table 2.2** provides logistic regression analysis of the relationship between CCR and CMM based on the clinic data. In the analysis of clinical data, CCR_per_TIR across all models (Model 1: unadjusted; Model 2: age and gender; Model 3: age, gender, location, marital and HTN; Model 4: fully adjusted) had an OR of 1.00 (95% CI: 0.97–1.03, P > 0.05). Similarly, for CCR layering, neither Q2 nor Q3 showed any significant associations across the models (P > 0.05).

**Table 2.1:** Logistic regression analysis of the relationship between CCR and CMM based on the CHARLS database.

CCR		Model1	P	Model2	P	Model3	P	Model4	P
CCR_per_TIR		0.99[0.99,1.00]	<0.001	1.00[0.99,1.00]	0.001	1.00[0.99,1.00]	0.01	1.00[1.00,1.00]	0.353
CCR	Q1	ref		ref		ref		ref	
	Q2	0.98[0.97,0.99]	<0.001	0.98[0.97,0.99]	<0.001	0.99[0.98,1.00]	0.005	0.99[0.98,1.00]	0.186
	Q3	0.97[0.96,0.98]	<0.001	0.98[0.97,0.99]	<0.001	0.98[0.97,0.99]	<0.001	0.99[0.98,1.00]	0.109

**Table 2.2:** Logistic regression analysis of the relationship between CCR and CMM based on the clinic data.

CCR		Model1	P	Model2	P	Model3	P	Model4	P
CCR_per_TIR		1.00[0.97,1.03]	0.952	1.00[0.97,1.03]	0.925	1.00[0.97,1.03]	0.928	1.00[0.97,1.03]	0.934
CCR	Q1	ref		ref		ref		ref	
	Q2	0.96[0.86,1.07]	0.491	0.99[0.89,1.01]	0.856	1.00[0.90,1.11]	0.969	0.98[0.88,1.1]	0.772
	Q3	0.95[0.85,1.06]	0.329	0.97[0.86,1.01]	0.648	0.97[0.86,1.09]	0.648	0.96[0.85,1.08]	0.525

CMM, cardiometabolic multimorbidity; METS-IR, Metabolic score for insulin resistance; CCR, creatinine-to-cystatin C ratio; CI, confidence interval; CCR_per_TIR, CCR value standardized by tertile interval width.

Charls data: Model 1: unadjusted. Model 2: adjusted for age and gender. Model 3: age, gender, location, marital status, smoking, drinking, HTN, CLD and CA. Model 4: age, gender, location, marital status, smoking, drinking, HTN, CLD, CA, WBC, HbA1c and TC.

Clinic data: Model 1: unadjusted. Model 2: adjusted for age and gender. Model 3: age, gender, location, marital status, and HTN. Model 4: adjusted for age, gender, location, marital status, HTN, WBC and LDL.

The logistic regression model for the latter data is the same as those models.

### 4.3 Impacts of METS-IR on CMM occurrence

In the CHARLS database analysis (**Table 3.1**), for the continuous metric METS-IR_per_TIR, Model 1 yielded an OR of 1.03 (95% CI: 1.02–1.03, P < 0.001), indicating a significant positive link with CMM. After adjusting for age and gender (Model 2), the OR remained elevated at 1.03 (95% CI: 1.03–1.03, P < 0.001), suggesting independence from basic demographics. In Model 3 with further adjustment, which included location, marital status, smoking, drinking, hypertension (HTN), chronic lung disease (CLD) and cancer (CA), the association remained significant at an OR of 1.02 (95% CI: 1.02–1.02, P < 0.001), albeit with a slight attenuation in effect size. After comprehensive adjustment for age, gender, location, marital status, HTN, white blood cell count (WBC), glycated hemoglobin (HbA1c), and total cholesterol (TC) in Model 4, the OR was 1.01 (95% CI: 1.01–1.02, P < 0.001), confirming the persistence of the significant association. For METS-IR stratified by quantiles (Q1 as reference), Q2 in Model 1 had an OR of 1.01 (95% CI: 1.00–1.02, P = 0.006) with variable significance, while Q3 in Model 1 presented an OR of 1.07 (95% CI: 1.06–1.08, P < 0.001), maintaining consistency across models and indicating graded risk.

**Table 3.1:** Logistic regression analysis of the relationship between METS-IR and CMM based on the CHARLS data.

METS-IR		Model1	P	Model2	P	Model3	P	Model4	P
METS-IR_per_TIR		1.03[1.02,1.03]	<0.001	1.03[1.03,1.03]	<0.001	1.02[1.02,1.02]	<0.001	1.01[1.01,1.02]	<0.001
METS-IR	Q1	ref		ref		ref		ref	
	Q2	1.01[1.00,1.02]	0.006	1.02[1.01,1.03]	<0.001	0.99[0.99,1.00]	0.052	1.00[0.99,1.01]	0.601
	Q3	1.07[1.06,1.08]	<0.001	1.08[1.07,1.09]	<0.001	1.00[0.99,1.01]	<0.001	1.03[1.02,1.04]	<0.001

In clinical data analysis (**Table 3.2**), for continuous METS-IR_per_TIR, all models showed significant positive associations with ORs ranging from 1.06 to 1.08. For stratified METS-IR, Q2 in Model 1 had an OR of 1.11 (95% CI: 1.10–1.24, P = 0.059) and Q3 in Model 1 displayed an OR of 1.19 (95% CI: 1.06–1.32, P = 0.002), indicating a significant correlation between the models and strengthening the dose-response relationship. Overall, METS-IR was significantly associated with increased CMM risk in both datasets.

**Table 3.2:** Logistic regression analysis of the relationship between METS-IR and CMM based on the clinic data.

METS-IR		Model1	P	Model2	P	Model3	P	Model4	P
METS-IR_per_TIR		1.07[1.04,1.11]	<0.001	1.08[1.04,1.11]	<0.001	1.06[1.03,1.10]	<0.001	1.06[1.03,1.10]	<0.001
METS-IR	Q1	ref		ref		ref		ref	
	Q2	1.11[1.0,1.24]	0.059	1.17[1.05,1.30]	0.005	1.14[1.02,1.27]	0.02	1.11[1.00,1.24]	0.051
	Q3	1.19[1.06,1.32]	0.002	1.22[1.10,1.36]	<0.001	1.16[1.04,1.30]	0.008	1.14[1.02,1.27]	0.024

METS-IR_per_TIR, METS-IR value standardized by tertile interval width.

### 4.4 Association between combinations of METS-IR and CCR tertile groups and CMM

**Figure 2:**
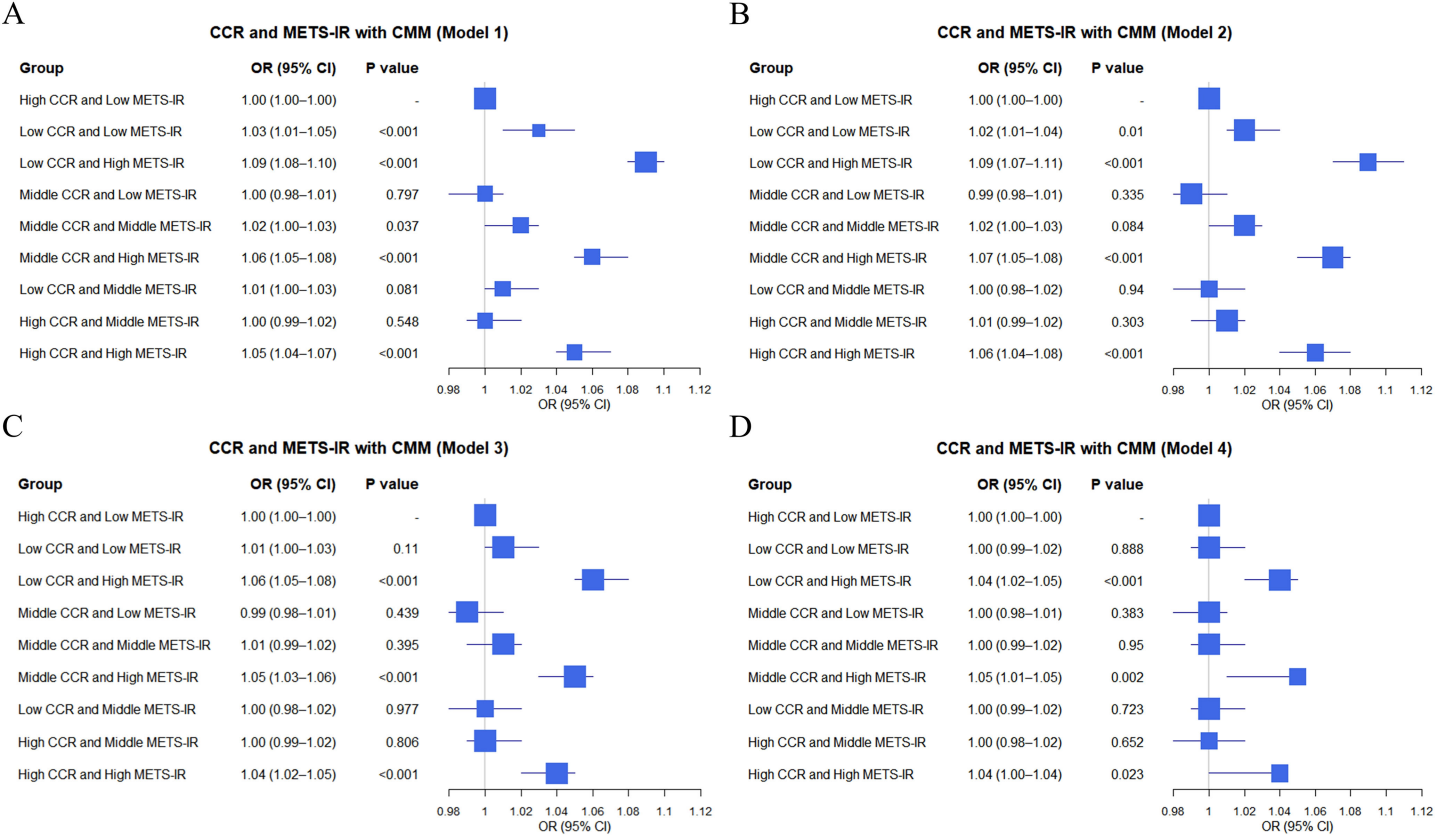
Logistic regression analysis of the relationship between combinations of CCR and METS-IR and CMM in different models based on CHARLS data **(A–D)**.

This cross-sectional study explored the impact of combinations of METS-IR and CCR tertile groups on CMM using data from the CHARLS database and clinical sources. In the CHARLS database (**Figure 2**), various combinations showed distinct effects on CMM across models with “High CCR and Low METS-IR” as the reference. For instance, “Low CCR and High METS-IR” had a significantly increased risk of CMM in Model 1 (OR = 1.09, 95% CI: 1.08–1.10, P < 0.001) (**Figure 2A**) and remained significant in Model 2 (OR = 1.09, 95% CI: 1.07–1.11, P < 0.001) (**Figure 2B**). “Middle CCR and High METS-IR” also exhibited a heightened risk, with OR of 1.06 (95% CI: 1.05–1.08, P < 0.001) in Model 1 (**Figure 2A**) and OR of 1.07 (95% CI: 1.05–1.08, P < 0.001) in Model 2 (**Figure 2B**). Conversely, some combinations like “Middle CCR and Low METS-IR” showed non-significant associations in multiple models.

**Figure 3:**
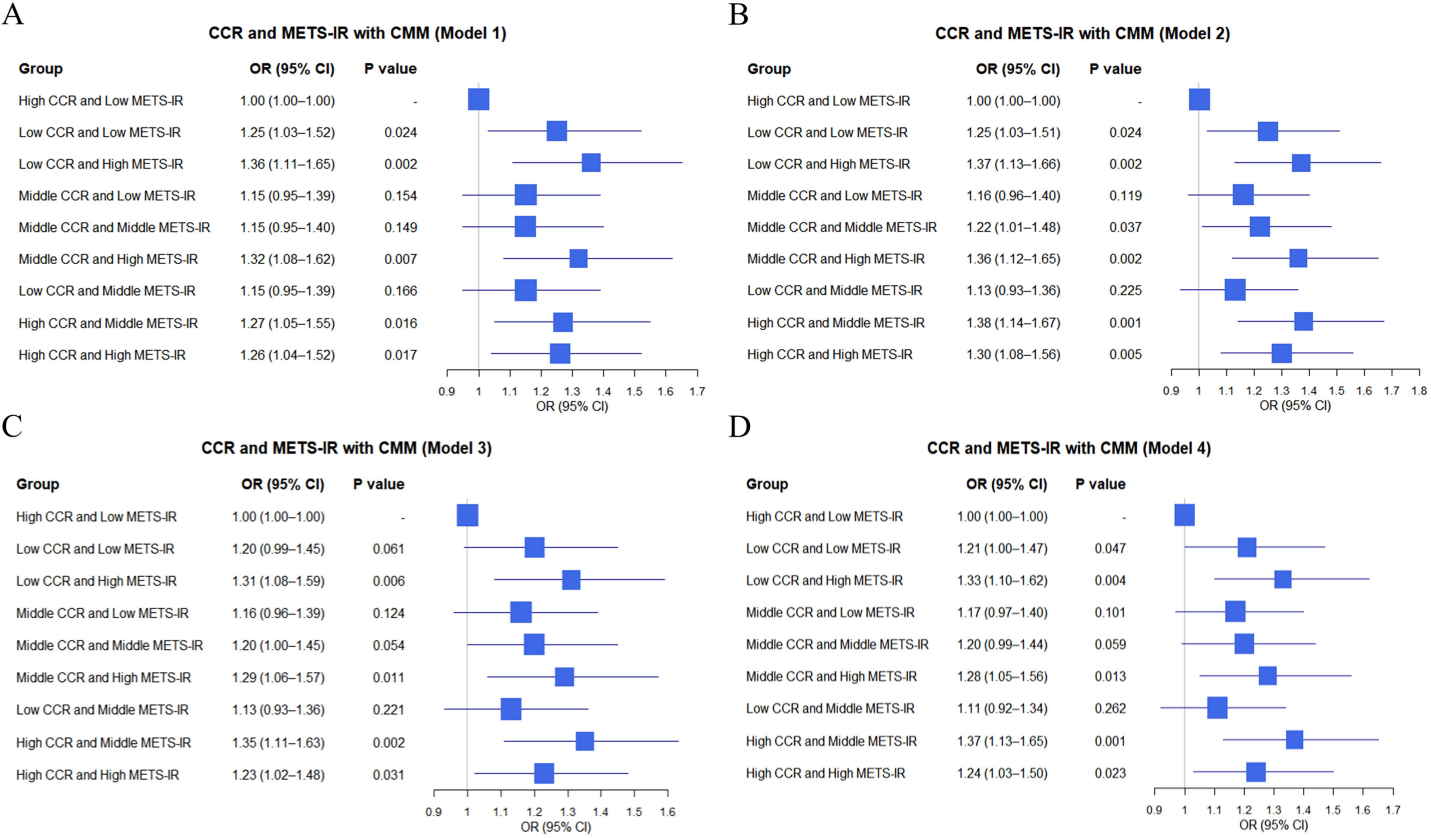
Logistic regression analysis of the relationship between combinations of CCR and METS-IR and CMM in different models based on clinic data **(A–D)**.

In clinical data (**Figure 3**), similar analyses were conducted. For instance, “Low CCR and High METS-IR” was associated with an elevated CMM risk, with OR of 1.36 (95% CI: 1.11–1.65, P = 0.002) in Model 1 (**Figure 3A**) and OR of 1.37 (95% CI: 1.13–1.66, P = 0.002) in Model 2 (**Figure 3B**). “Middle CCR and High METS-IR” also demonstrated a significant impact, with OR of 1.32 (95% CI: 1.08–1.62, P = 0.007) in Model 1 (**Figure 3A**) and OR of 1.36 (95% CI: 1.12–1.65, P = 0.002) in Model 2 (**Figure 3B**). Overall, the combinations of METS-IR and CCR tertile groups were associated with CMM occurrence, with variations in effect sizes and significance across different models in both datasets.

### 4.5 Association between MRII and CMM

In the CHARLS database (**Table 4.1**), distinct effects on CMM were observed across models for different quartile groups, with the Q1 group of “MRII” as the reference. For the Q2 group, Model 1 yielded an OR of 1.00 (95% CI: 0.99–1.01], P = 0.838), and Model 2 showed an OR of 1.01 (95% CI: 1.00–1.02, P = 0.045). The Q3 group also exhibited elevated CMM risk. Model 1 had an OR of 1.02 (95% CI: 1.00–1.03, P = 0.005), and Model 2 presented an OR of 1.03 (95% CI: 1.02–1.04, P < 0.001). The Q4 group demonstrated a significantly increased risk, with Model 1 showing an OR of 1.03 (95% CI: 1.02–1.04, P < 0.001) and Model 2 an OR of 1.05 (95% CI: 1.04–1.06, P < 0.001). In clinical data (**Table 4.2**), using the Q1 group as the reference, both the Q2 group and the Q3 group showed non-significant associations in the four models. However, the Q4 group consistently indicated a heightened CMM risk. Model 1 reported an OR of 1.22 (95% CI: 1.07–1.38, P = 0.001), and Model 2 showed an OR of 1.22 (95% CI: 1.08–1.38, P = 0.002). Overall, quartile groupings of the “MRII” were associated with CMM occurrence. In the CHARLS database, Q3 and Q4 groups were linked to increased CMM risk across models. In clinical data, the Q4 group consistently predicted a higher CMM risk.

**Table 4.1:** Logistic regression analysis of the relationship between MRII and CMM based on the CHARLS data.

MRII		Model		Model2		Model3		Model4	
MRII	Q1	ref		ref		ref		ref	
	Q2	1.00[0.99,1.01]	0.838	1.01[1.00,1.02]	0.045	1.00[0.99,1.02]	0.391	1.00[0.99,1.01]	0.51
	Q3	1.02[1.00,1.03]	0.005	1.03[1.02,1.04]	<0.001	1.02[1.01,1.03]	0.001	1.01[1.00,1.02]	0.02
	Q4	1,03[1.02,1.04]	<0.001	1.05[1.04,1.06]	<0.001	1.03[1.02,1.04]	<0.001	1.02[1.01,1.03]	0.001

**Table 4.2:** Logistic regression analysis of the relationship between MRII and CMM based on the clinical data.

MRII		Model1	P	Model2	P	Model3	P	Model4	P
MRII	Q1	ref		ref		ref		ref	
	Q2	1.07[0.94,1.21]	0.318	1.10[0.97,1.24]	0.133	1.08[0.96,1.22]	0.22	1.08[0.95,1.22]	0.23
	Q3	1.09[0.96,1.23]	0.2	1.10[1.07,1.25]	0.135	1.07[0.95,1.22]	0.282	1.06[0.94,1.20]	0.334
	Q4	1.22[1.07,1.38]	0.002	1.22[1.08,1.38]	0.002	1.17[1.03,1.33]	0.016	1.17[1.03,1.33]	0.015

### 4.6 The RCS curves of METS-IR, CCR, and their combination for CMM

**Figure 4:**
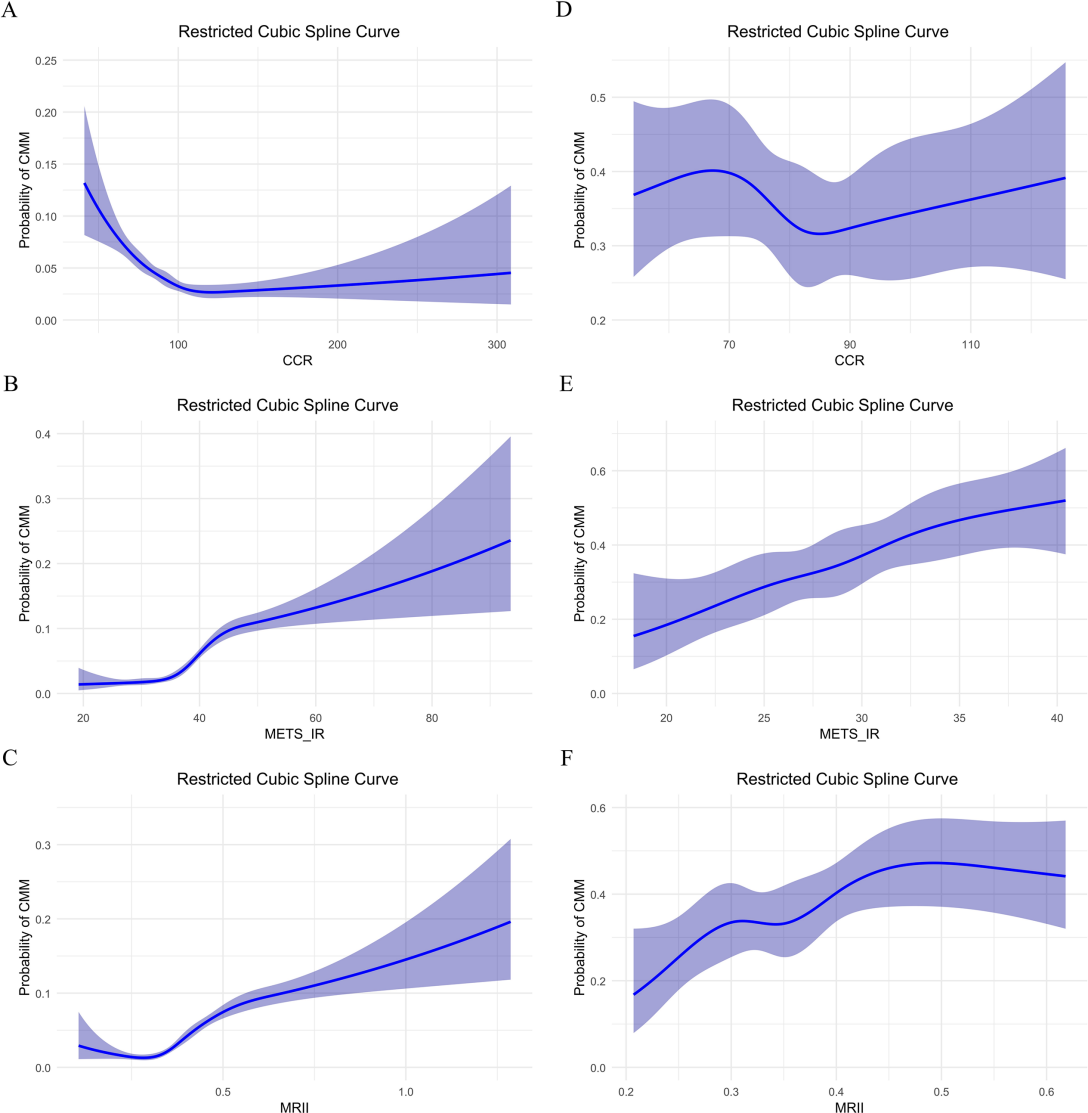
Restricted cubic spline curve analysis of the relationship between CCR, METS-IR, MRII and the probability of CMM: comparison of two data sets. **(A–C)** In CHARLS data, CCR, METS-IR or MRII effect on probability of CMM, respectively. **(D–F)** In clinical data, CCR, METS-IR or MRII effect on probability of CMM, respectively.

In the CHARLS database, CCR showed a U-shaped association with CMM including the high probability (0.12–0.20) at low CCR (near 0), a nadir (0.03–0.05) in the interval of 80–120 (validated via piece-wise regression), and renewed increase beyond a CCR of 120 (reaching 0.12–0.13 at a CCR of 300) (**Figure 4A**). METS-IR demonstrated a positive, approximately linear relationship with CMM risk, with probabilities ranging from 0.01–0.10 at values of 20–40, and rising to 0.25–0.30 at a value of 100 (**Figure 4B**). In contrast, the MRII exhibited a nonlinear “decrease-then-increase” pattern, confirming a synergistic relationship between the two factors (**Figure 4C**). In the clinical data, the relationship between CCR and CMM risk was a nonlinear pattern with the lowest risk (0.30–0.35) observed at CCR values of 70–90, and higher risk outside this range (**Figure 4D**). Leveraging Youden’s index, we identified 129 as the optimal high-risk threshold for CCR, with a specificity of 0.993 and a sensitivity of 0.032 (maximum Youden’s index = 0.025). In contrast, METS-IR exhibited an overall increasing trend, rising from 0.20–0.30 at values of 20–40 to between 0.30–0.60 beyond 40 (**Figure 4E**). Via Youden’s index, the optimal high-risk threshold for METS-IR was determined as 27.474, with a sensitivity of 0.721, specificity of 0.463, and a maximum Youden’s index of 0.184. The MRII also demonstrated a complex, fluctuating nonlinear association (**Figure 4F**).

**Figure 5:**
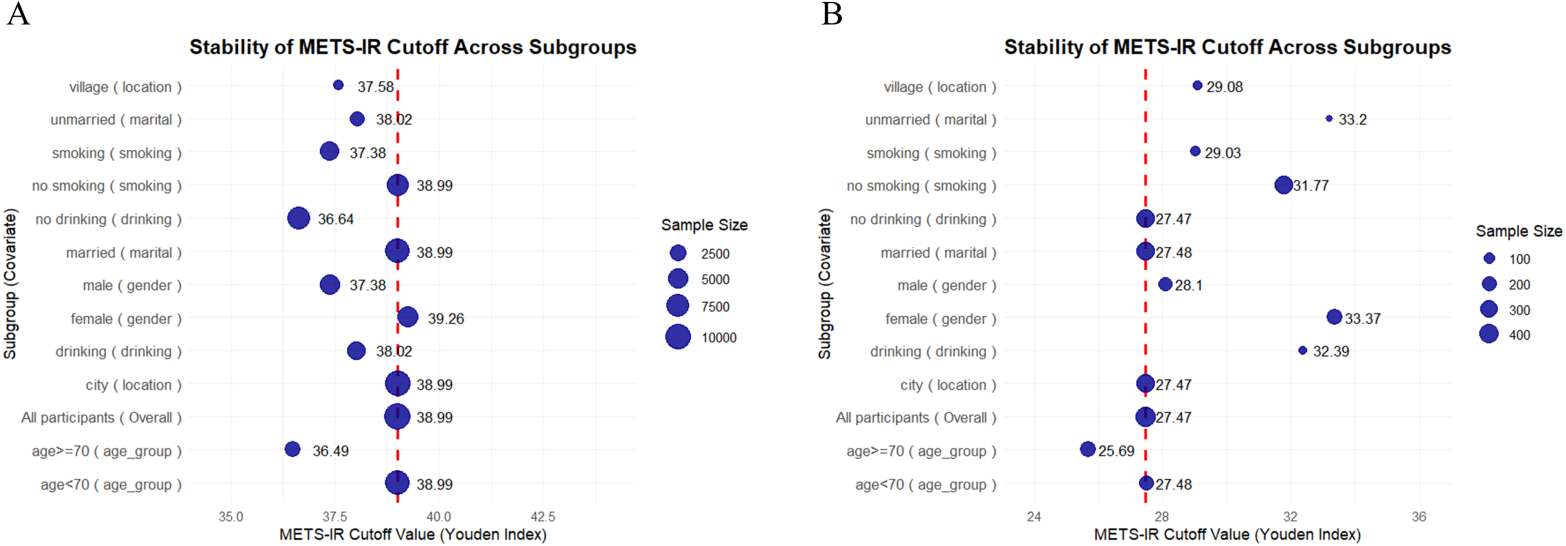
Scatter plot of METS-IR cutoff stability across subgroups (weighted by sample size) in CHARLS data **(A)** and clinical data **(B)**. The blue dots represent the METS-IR cutoff values (derived from the Youden’s index) for different subgroups. The size of the blue dots corresponds to the sample size of each subgroup. The red dashed line denotes the overall METS-IR cutoff value for the entire population.

Furthermore, we assessed the stability of METS-IR cut-off values across subgroups by utilizing the Youden’s index (**Figure 5**). In the clinical dataset (**Figure 5B**), the overall METS-IR threshold was determined to be 27.47, demonstrating relative stability within subgroups such as urban residents, married individuals, and non - drinkers (ranging from 27.47 to 28.1). Conversely, higher cut-off points were observed in subgroups including females (33.37), rural residents, drinkers, unmarried individuals, and smokers. Additionally, the subgroup of individuals aged 70 years or older (25.69) had a lower cut-off point. In contrast, the CHARLS dataset (**Figure 5A**) presented a substantially higher overall threshold (38.99), with subgroup thresholds spanning from 36.49 to 39.26. Specifically, females (39.26) had a higher threshold compared to males (37.38). The subgroup of individuals younger than 70 years (38.99) was consistent with the overall threshold, while the subgroup of those aged 70 years or older (36.49) had a lower threshold. Moreover, urban residents and married individuals had thresholds in line with 38.99, whereas rural residents, unmarried individuals, and the subgroup of those aged 70 years or older had lower thresholds.

### 4.7 Predictive value of METS-IR, CCR, and MRII for CMM

**Figure 6:**
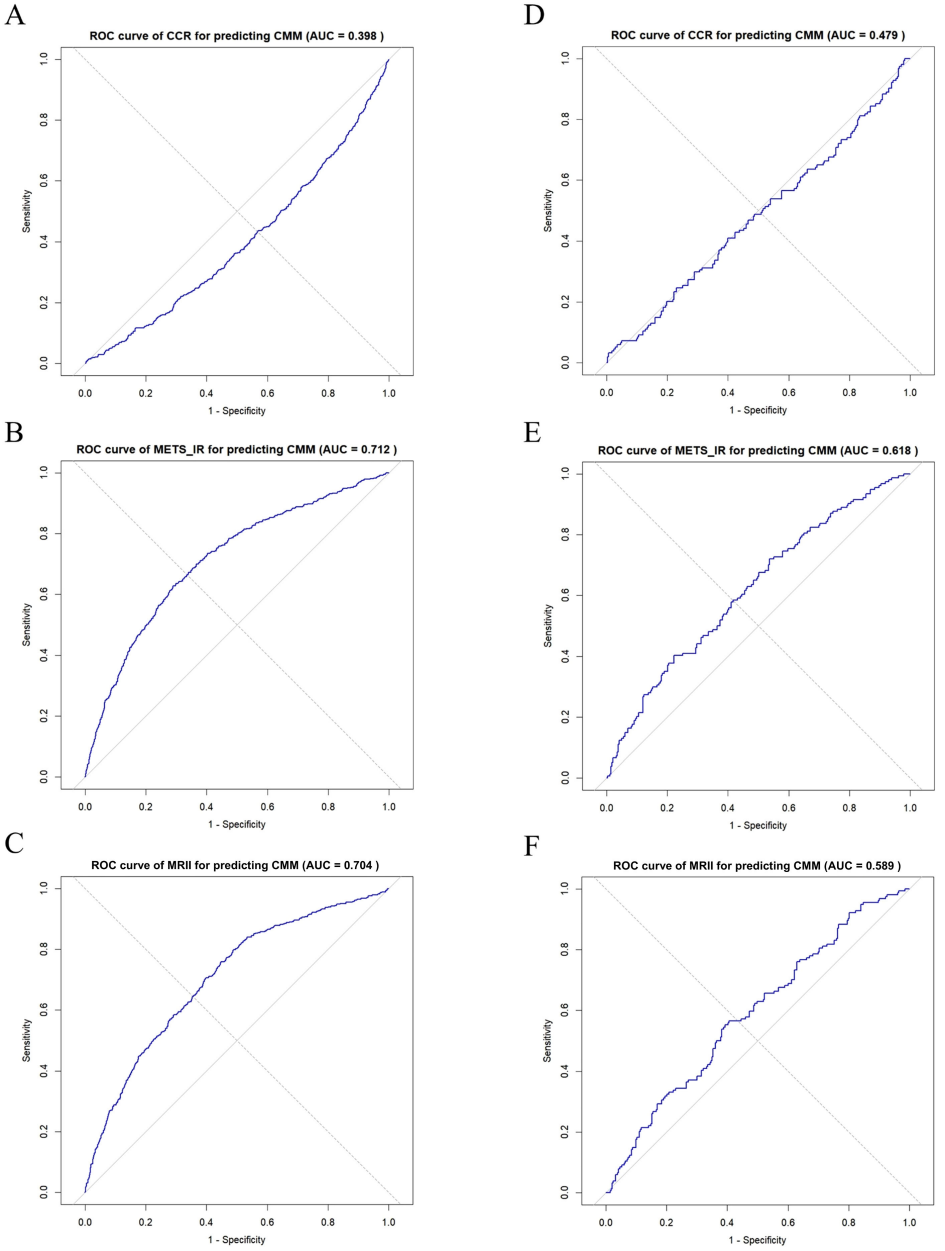
ROC curve analysis of CCR, METS-IR, and MRII for predicting CMM: comparison between two data sets. **(A–C)** In CHARLS data, ROC curve of CCR, METS-IR or MRII for predicting CMM, respectively. **(D–F)** In clinical data, ROC curve of CCR, METS-IR or MRII for predicting CMM, respectively.

In the CHARLS database (**Figures 6A–C**), receiver operating characteristic (ROC) curve analyses (**Figure 6B**) showed that METS-IR exhibited moderate-to-good discriminative ability for CMM (area under the curve [AUC] = 0.712), while the MRII demonstrated predictive potential (AUC = 0.704) (**Figure 6C**), which also indicated that the MRII adds discriminatory power (albeit slightly less than METS-IR alone). However, CCR had limited predictive performance (AUC = 0.398) as a standalone marker (**Figure 6A**). In clinical data (**Figures 6D–F**), METS-IR retained modest predictive value for CMM (AUC = 0.618) to assist clinical risk stratification (**Figure 6E**). The MRII showed moderate-to-poor discriminative ability (AUC = 0.589), highlighting the need to optimize multi-marker combination models (**Figure 6F**). In addition, CCR demonstrated poor predictive performance (AUC = 0.479, near random prediction) and is not recommended as a standalone predictor (**Figure 6D**).

### 4.8 Subgroup analysis of the effects of CCR, METS-IR, and MRII on CMM: comparison between CHARLS and clinical data

**Figure 7:**
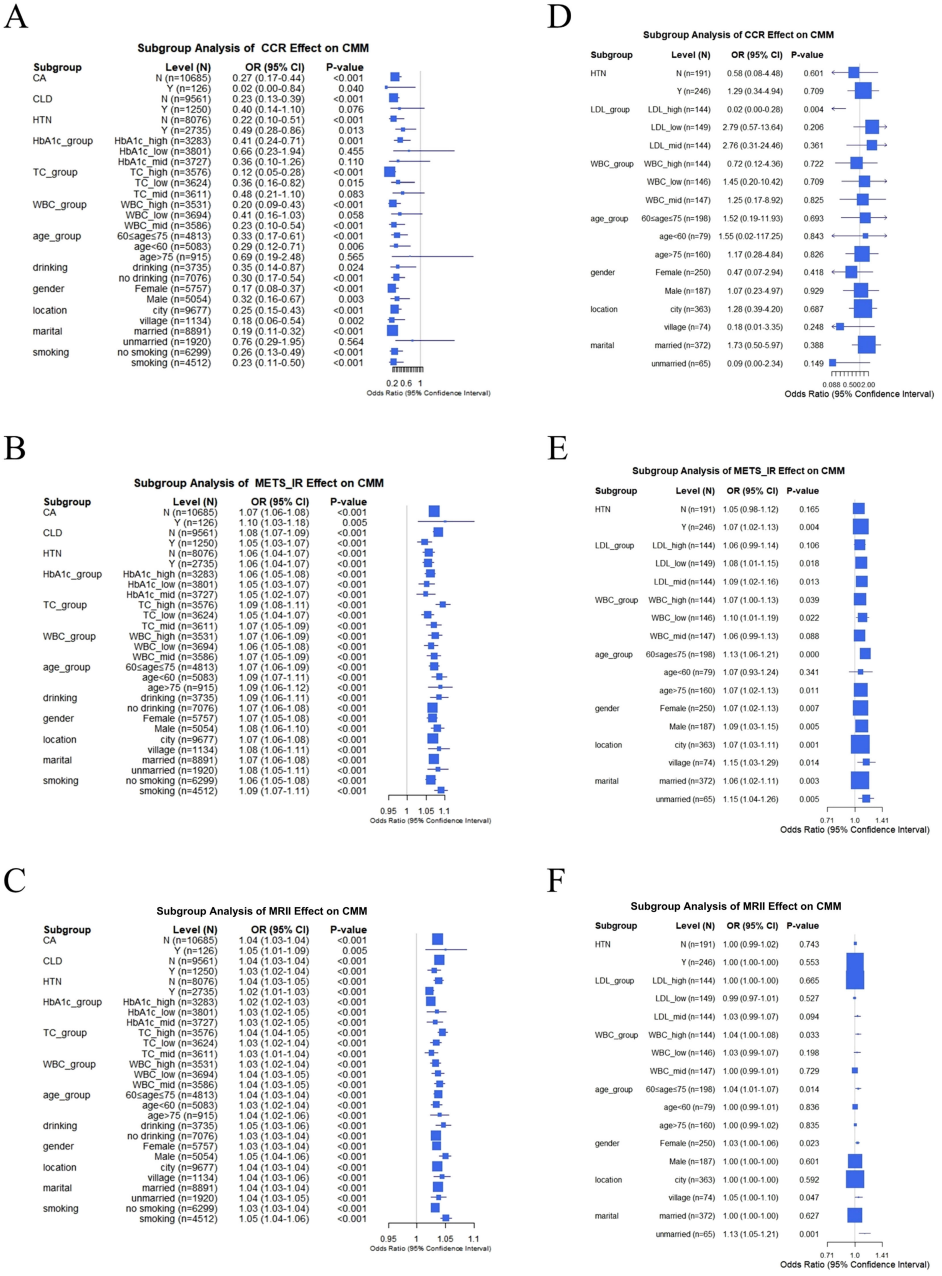
Subgroup analysis of the effects of CCR, METS-IR, and MRII on CMM: comparison between CHARLS and clinical data. **(A–C)** In CHARLS data, subgroup analysis of CCR, METS-IR or MRII effect on CMM, respectively. **(D–F)** In clinical data, subgroup analysis of CCR, METS-IR or MRII effect on CMM, respectively.

In the CHARLS database, CCR had significantly heterogeneous effects on CMM across subgroups (**Figure 7A**). In the cancer (CA) subgroup, the odds ratio (OR) of CCR for CMM was 0.27 (95% CI: 0.17–0.44; P < 0.001) in individuals without cancer (N, n = 10,685) and 0.02 (95% CI: 0.00–0.84; P = 0.040) in those with cancer (Y, n = 126). In the chronic lung disease (CLD) subgroup, the OR was 0.23 (95% CI: 0.13–0.39, P < 0.001) in individuals without CLD (N, n = 9561), and 0.40 (95% CI: 0.14–1.10, P = 0.076) in individuals with CLD (Y, n = 1250), indicating protective effect of CCR on CMM varies by disease background. Subgroup analyses of biochemical indicators and demographics revealed modifying effects. For example, different age groups exhibited varying CCR effects (< 60 years, OR = 0.29, 95% CI: 0.12–0.71, P = 0.006; 60–75 years, OR = 0.33, 95% CI: 0.17–0.61, P < 0.001; > 75 years, OR = 0.69, 95% CI: 0.19–2.48, P = 0.565). This result indicated a positive association between CCR and CMM. As shown in the CA subgroup (**Figure 7B**), METS-IR was significantly associated with higher CMM odds in both non-cancer (OR = 1.07, 95% CI: 1.06–1.08, P < 0.001) and cancer (OR = 1.10, 95% CI: 1.03–1.18, P = 0.005) participants. This positive association was consistently observed in various subgroups (such as CLD, total cholesterol levels, and age), although the magnitude of risk elevation varied. Notably, MRII also demonstrated significance across these subgroups (**Figure 7C**). Subgroup analyses of clinical data revealed more complex relationships among CCR, METS-IR, and MRII on CMM (**Figures 7D–F**). For example, in the HTN subgroup, CCR was not significantly associated with CMM in either normotensive (OR = 0.58, 95% CI: 0.08–4.48, P = 0.601) or hypertensive individuals (OR = 1.29, 95% CI: 0.34–4.94, P = 0.709) (**Figure 7D**). However, in individuals with HTN, METS-IR exhibited a significant odds ratio (OR) of 1.07 (95% CI: 1.02–1.13, P = 0.004), highlighting the modifying role of disease status (**Figure 7E**). In conclusion, the associations of METS-IR, CCR, and MRII with CMM are significantly modulated by subgroup characteristics.

## 5 Discussion

In this study, we systematically investigated the relationships between the METS-IR, CCR, and CMM using both the CHARLS database and clinical data, yielding comprehensive findings with implications for clinical practice and public health. Baseline analyses consistently demonstrated that across both datasets, individuals with CMM were older, had a higher BMI, a higher prevalence of metabolic risk factors (e.g., hypertension, diabetes), and elevated METS-IR compared to non-CMM groups.

METS-IR integrates factors closely related to insulin resistance and metabolic abnormalities, including fasting blood glucose, triglycerides and HDL-C, which not only reflect metabolic health but also correlate with cardiovascular risk factors like hypertension, obesity, and dyslipidemia (17). Zhou et al. found that there is a significant positive and nonlinear relationship between METS-IR and CMM, regardless of adjusting for other confounding factors (38), which is consistent with our research findings. Our logistic regression analysis revealed that METS-IR emerged as a robust and independent risk factor for CMM in both populations. Continuous METS-IR showed significant positive associations with CMM across all adjustment models. Furthermore, tertile-stratified analysis confirmed a graded increase in risk, with the highest tertile (Q3) consistently linked to elevated odds ratios, indicating a clear dose-response relationship. Previous studies, through cross-sectional and longitudinal analyses, have found that higher METS-IR independently predicts hypertension incidence and prevalence in older Chinese adults (39). A prospective cohort study in the 51st Regiment of the Third Division of the Xinjiang Production and Construction Corps linked elevated baseline insulin resistance surrogates and their long-term trajectories to high CVD risk in rural Xinjiang (40). In addition, data from the National Health and Nutrition Examination Survey (2009-2018) showed that METS-IR was significantly positively correlated with the prevalence of type 2 diabetes (28). Antonio Aznar Esquivel et al. linked METS-IR to cardiovascular event risk factors (41). Therefore, consistent with previous extensive research, our findings validated the effectiveness of METS-IR in predicting cardiovascular disease risk.

In contrast, the association between CCR and CMM is more context-dependent. In the CHARLS database, CCR was inversely associated with CMM in unadjusted and partially adjusted models, though significance diminished in fully adjusted models. While in clinical data, no significant associations were observed for continuous CCR or its tertile strata, suggesting confounding by other factors or population-specific characteristics. However, the context-dependent association of CCR with CMM may be explained by several factors. Firstly, the population setting is critical. The measure may reflect early, subclinical dysregulation in a community cohort (CHARLS) but be confounded by acute illness in a hospitalized clinical population. Furthermore, the substantially smaller sample size of the clinical dataset (n = 437 vs. n = 10,811) limits statistical power to detect modest effects. Lastly, residual confounding or effect modification by unmeasured metabolic factors may differentially influence the association across these distinct populations. Yulin Chen et al. showed that the increased CCR may predict CVD in older adults via cross-sectional and longitudinal studies of 10,614 and 6,726 community volunteers (37). Jang Yel Shin found low CCR independently associated with sarcopenia and severe carotid atherosclerosis (closely linked to stroke) in 1,577 type 2 diabetes patients (25). Another study linked lower CCR to increased new-onset CVD risk in middle-aged and older Chinese individuals, and restricted cubic splines showed a significant linear relationship between the sarcopenia index and CVD incidence (42). Honglin Sun et al. established the weight-adjusted muscle mass index as a reliable predictor of CMM onset and progression in Chinese middle-aged and older adults, particularly among women (36). The results above align with previous research confirming the connection between CCR and CMM. While in clinical data, CCR tertiles Q2 and Q3 showed no significant associations across models, which highlighted the complex interplay between metabolic indices and cardiometabolic health.

Based on existing evidence, plausible pathways underlying the relationship among METS-IR, CCR and CMM are as follows. Firstly, CCR, a reliable marker for muscle mass and sarcopenia (20, 21), links sarcopenia to metabolic abnormalities (oxidative stress, chronic inflammation) (43–45), which drive CMM components (e.g., heart disease, diabetes) by damaging vascular endothelium and disrupting glucose/lipid metabolism (46, 47). Secondly, insulin resistance (IR), reflected by METS-IR, drives CMM. IR accelerates muscle loss centered around muscles (48–50), forming a vicious cycle of low CCR (reduced glucose processing). In the vasculature, IR directly promotes atherosclerosis and dyslipidemia (51). Additionally, endothelial SGK-1 activation has been identified as a mediator of IR-induced arterial stiffness (52). Thirdly, “High METS-IR and Low CCR” creates a self-reinforcing cycle. IR impairs muscle via inflammation and mitochondrial dysfunction (49, 50), while muscle loss worsens IR and amplifies CMM risk. These pathways, including oxidative stress, inflammation, insulin signaling, and muscle-metabolism crosstalk, provide a biological framework for the findings.

We found that lower CCR can reduce CMM risk and higher METS-IR increases it based on CHARLS and clinical data. The combined effects of METS-IR and CCR further clarified their interactive role in CMM. Tertile combination analyses revealed that “Low CCR and High METS-IR” consistently associated with increased CMM risk in both datasets. Even after multivariable adjustment, the MRII showed a similar trend that higher quartiles (especially Q4) are associated with higher CMM probabilities, indicating that their synergistic effect better captures risk than using any single index alone. Restricted cubic spline (RCS) curves illuminated nonlinear relationships. METS-IR showed a positive linear association with CMM in both datasets, while CCR exhibited a U-shaped relationship in CHARLS and a fluctuating nonlinear pattern in clinical data. Notably, the identified CCR ‘low-risk interval’ (80–120) in the CHARLS cohort may lack generalizability to hospitalized clinical patients. In the RCS curves, the median CCR in the clinical sample is close to the lower bound of this interval, likely due to acute illness effects on creatinine and cystatin C levels in hospitalized individuals. Thus, this interval should be interpreted with caution when applied to such clinical populations.

Predictive value assessments via ROC curves indicated that METS-IR had moderate-to-good discriminative ability for CMM (AUC 0.712 in CHARLS, 0.618 in clinical data), outperforming CCR (AUC 0.479 in clinical data) and the MRII, which supports its potential as a practical screening tool. Meanwhile, the subgroup analyses revealed significant heterogeneity. Age, cancer status, chronic lung disease, and hypertension altered the relationship between CCR, METS-IR, and CMM, emphasizing the necessity of personalized risk assessment.

Overall, these findings confirm that METS-IR is a consistent and influential predictive factor for CMM. Although CCR is not as robust, its interaction with METS-IR affects risk, especially when used in combination. Differences between CHARLS and clinical datasets likely reflect variations in population characteristics (e.g., age, comorbidities).With the change of lifestyle, the incidence rate of obesity and metabolic syndrome increases, thus increasing cardiovascular risk (39). These results deepen the understanding of cardiac metabolic risk stratification, indicating that comprehensive METS-IR and CCR assessment can enhance CMM risk prediction and provide information for targeted prevention strategies. However, further longitudinal studies are warranted to confirm causal relationships and explore underlying mechanisms, as these findings provide a theoretical basis for early identification of high-risk groups and personalized CMM prevention and treatment.

These findings inform clinical practice by prioritizing METS-IR as a robust first-line tool for CMM risk stratification. It demonstrates a clear dose-response relationship, moderate-to-good discriminative ability, and elevated risk beyond a threshold of 40, warranting routine monitoring in middle-aged/older adults and those with metabolic risk factors. The utility of CCR depends on specific circumstances. It is valuable for early risk identification in community environments, but its reliability for hospitalized patients is low due to acute pathology or limited sample size. The integration of CCR and METS-IR indicators can enhance risk prediction, especially for the “Low CCR and High METS-IR” phenotype, indicating a synergistic effect of metabolic renal dysfunction. Personalized assessment should account for modifiers like age, hypertension, and chronic lung disease to guide targeted interventions (e.g., lifestyle or pharmacotherapy) for high-risk groups, while future work should address the limitations of cross-sectional data and pursue standardized measurements in diverse cohorts.

This study demonstrates key strengths. Firstly, our study integrates two different data sources, a large CHARLS cohort representing the middle-aged and elderly population in China and real-world clinical data, improving the universality of the dataset, and validating the research results. Secondly, our study employed a comprehensive analytical approach, including multi-model logistic regression with rigorous adjustment, quantile stratification, combined effect assessments, RCS curves for nonlinear relationships, ROC-based predictive analyses, and subgroup evaluations. This approach enabled an in-depth investigation into the roles of METS-IR and CCR in CMM. Thirdly, focusing on both individual indices and their interactions offers novel insights into synergistic metabolic-renal mechanisms underlying CMM, refining cardiometabolic risk stratification.

However, there are several limitations in our current study. Firstly, as a cross-sectional study, it is inherently limited in establishing causal links. Longitudinal studies are necessary to elucidate these temporal relationships. Secondly, the observed discrepancies between CHARLS and clinical datasets, such as the weaker associations for CCR in clinical data, may reflect population differences (e.g., in age or comorbidity profiles) or heterogeneity in data collection procedures, both of which could act as confounders. Finally, residual confounding caused by unmeasured factors, such as diet, physical activity, and medication use, cannot be eliminated.

## 6 Conclusion

METS-IR is a robust independent predictor of CMM in both community and hospitalized populations, while CCR’s role is context-dependent. Their interaction enhances CMM risk stratification, highlighting the value of concurrent assessment of metabolic and muscle-renal status for CMM prevention and personalized risk management.

## Data Availability

Publicly available datasets were analyzed in this study. This data can be found here: http://charls.pku.edu.cn/en.
