# Supplementary material for: Cardiometabolic multimorbidity in relation to the metabolic score for insulin resistance and creatinine-to-cystatin C ratio in a middle-aged and aged population
Source: Front Endocrinol (Lausanne). 2025 Dec 1;16:1694959. doi: 10.3389/fendo.2025.1694959 (PMC12703203; doi:10.3389/fendo.2025.1694959)
Supplement: Supplementary file 1 [file Table1.docx]

For the CHARLS dataset: The Vuong test yielded a z-score of 4.17 and a p-value < 0.001, indicating that the MRII model is statistically significantly better in fit than the CCR×METS-IR model.For the clinical data: The Vuong test p-values for both models are > 0.05 (0.901 for MRII and 0.099 for CCR×METS-IR), meaning there is no statistically significant difference in fit between the two models.

S1. Model fit comparison table for CHARLS dataset (AIC, Vuong Test)

|  | AIC | P-value | Z-score |
| --- | --- | --- | --- |
| MRII | 3651.34 | <0.001 | 4.17 |
| CCR×MRTS-IR | 3802.63 | 1 |  |

S2. Model fit comparison table for clinical data (AIC, Vuong Test)

|  | AIC | P-value | Z-score |
| --- | --- | --- | --- |
| MRII | 570.72 | 0.901 | -1.29 |
| CCR×MRTS-IR | 564.10 | 0.099 |  |

AIC (Akaike Information Criterion) measures model fit and complexity, with a lower value indicating a better model. P-value from the Vuong test determines if the difference in fit between two models is statistically significant (p < 0.05). Z-score is the test statistic for the Vuong test, where |z| > 1.96 typically corresponds to a significant p-value, indicating one model fits significantly better than the other.
